# Designing Insurance to Promote Use of Childhood Obesity Prevention Services

**DOI:** 10.1155/2013/379513

**Published:** 2013-04-09

**Authors:** Kimberly J. Rask, Julie A. Gazmararian, Susan S. Kohler, Jonathan N. Hawley, Jenny Bogard, Victoria A. Brown

**Affiliations:** ^1^Rollins School of Public Health of Emory University, Atlanta, GA 30322, USA; ^2^Alliance for a Healthier Generation, New York, NY 10005, USA

## Abstract

Childhood obesity is a recognized public health crisis. This paper reviews the lessons learned from a voluntary initiative to expand insurance coverage for childhood obesity prevention and treatment services in the United States. In-depth telephone interviews were conducted with key informants from 16 participating health plans and employers in 2010-11. Key informants reported difficulty ensuring that both providers and families were aware of the available services. Participating health plans and employers are beginning new tactics including removing enrollment requirements, piloting enhanced outreach to selected physician practices, and educating providers on effective care coordination and use of obesity-specific billing codes through professional organizations. The voluntary initiative successfully increased private health insurance coverage for obesity services, but the interviews described variability in implementation with both best practices and barriers identified. Increasing utilization of obesity-related health services in the long term will require both family- and provider-focused interventions in partnership with improved health insurance coverage.

## 1. Background

The secular rise in obesity among American youth has been well documented. Among children and adolescents aged 2 to 19 years, 16.9% have a body mass index (BMI) for age at or above the 95th percentile and 31.7% have a BMI percentile at or above the 85th percentile [1, 2]. An estimated 60% of overweight 5- to 10-year-olds already have a cardiovascular disease risk factor or hyperinsulinemia and more than 20% have two or more risk factors [3, 4]. The impetus to address rising obesity rates is driven also by the trajectory of health care costs [5–7]. By recent estimates, the annual burden of obesity has risen to almost 10% of health care spending, amounting to $147 billion in 2008 [8]. 

With frequent access and opportunities to engage families, a 2005 Institute of Medicine (IOM) report concluded that physicians, nurses, dietitians, and other clinicians are in a key position to influence children and their parents to adopt healthy lifestyles [9–12]. Despite these recommendations, a national population-based survey found that obesity was diagnosed at only 18% of well-child visits for children with known obesity, and diet and activity counseling was documented for only 51% of known obese children [13]. One barrier is that few health insurance plans have covered the costs of obesity prevention or treatment, leaving providers with a disincentive to offer the services and families facing significant out-of-pocket expenses if it is offered [14]. The Alliance for a Healthier Generation, founded in 2005 by the American Heart Association and the William J. Clinton Foundation, collaborated with private health insurance companies and large self-insured employers nationally to promote health insurance coverage for the prevention, assessment, and treatment of childhood obesity. Insurers agree to pay for at least four follow-up visits with the child's primary care provider and at least four visits with a registered dietitian per year for children in the eighty-fifth percentile or higher of BMI for age. Over 2.5 million children are currently covered by participating organizations. Insurers and employers also agree to distribute annually at least two targeted communications to all eligible beneficiaries to educate and promote utilization as well as monitoring utilization by sharing administrative claims data yearly. The American Academy of Pediatrics (AAP) and the Academy of Nutrition and Dietetics (Academy) collaborated on the development of educational materials, provider webinars, care coordination resources, and family resource materials. The purpose of this study is to review the early experience, lessons learned, and key success factors from this novel initiative to address childhood obesity through insurance redesign. 

## 2. Methods

### 2.1. Overview

 This was a qualitative study to identify facilitators and barriers to increasing the provision of obesity-related counseling services through key informant interviews. Administrative claims data were used to measure the use of obesity-specific services by eligible children.

### 2.2. Key Informant Interviews

 In 2010-11, interviews were conducted with the implementation coordinators from all 16 insurers or employers who had offered the coverage for at least a year. The interview contained both closed and open-ended questions about the coverage offered, eligible population, roll-out process, and implementation strategies to explore successes, barriers, and lessons learned (Table 1). The questions were developed from a review of the literature and pilot tested with subject matter experts to ensure that key implementation domains were included and that the prompts elicited the desired information. After the first pilot interviews the guide was again revised. Dissemination strategies were described both in terms of the target audience and the intensity of outreach. Informants were also asked about their use of marketing and educational materials. Similar interviews were also held with participating staff from the AAP and Academy who represent pediatricians and registered dietitians, respectively. Interviews were tape-recorded with consent of the participants. The transcripts and hand written notes were reviewed by two team members (SK and JH) who used them to identify common themes and create a summary statement for each of the questions covered. 

Follow-up interviews were conducted with 10 organizations that had more than two-year experience offering the coverage. The goal of these interviews was to identify any changes made after the initial launch in administering the coverage, promoting it to beneficiaries and/or providers, and any new facilitators or barriers to implementation. The research team conducted a 30-minute phone interview with key informants using a tailored interview guide that was developed in a similar process to the initial guide. 

### 2.3. Administrative Claims Data Review

 Key informants were asked about changes in utilization of obesity-specific health care services and deidentified health plan administrative claims data were reviewed to monitor the use of obesity-related counseling codes. Overweight and obesity were identified by an ICD-9 diagnosis code of 278.XX or V85.5X.

## 3. Results

### 3.1. Baseline Interviews

 Ten of the sixteen organizations interviewed were health plans and six were employers (Table 2). The key findings are summarized in Table 3 and discussed in the following.

#### 3.1.1. Administrative Barriers to Offering New Coverage

 Most indicated that there has been little if any cost associated with offering the coverage although that had been an initial concern of the underwriters. Only two indicated that premiums increased as a direct result of offering the coverage.

#### 3.1.2. Claims Processing

For four insurers, claims processing for the obesity benefit are handled manually. Providers must collect BMI information and enter it manually into the tracking system. Six insurers have electronic systems to process and pay claims. Each insurer provides a set of billing codes (diagnosis, CPT, and HCPCS) for participating physicians to use for reimbursement. The billing codes are similar overall but unique due to each insurer's contractual requirements.

#### 3.1.3. Coverage Offered

 Six organizations allow a higher number of PCP or registered dietitian (RD) visits than the initiative required; three do not have any age restriction and five do not restrict the coverage based on BMI. Eleven allow direct billing by RDs but 5 required that counseling be provided by a physician only or by an RD working out of a physician's office. Thirteen required a copayment from families for PCP visits and ten required a co-payment for RD visits. Co-payments ranged from $20 to $75 per visit.

#### 3.1.4. Enrollment Process for Families

 Eleven organizations have no formal enrollment and/or pre-certification process required before receiving services. Members simply make an appointment with a PCP and/or dietitian of their choice. One employer requires members to call a toll free number to speak with an RD for eligibility determination. Two require that members have a referral to a dietitian. One requires that children participate in a disease management program in order to receive services. Two require that the child take an enrollment form to their initial baseline assessment with a physician. The physician then determines if they qualify for the counseling services and manually enters the information into an electronic tracking system. Organizations identified several barriers to engaging families. A lack of coordination between insurers and employers makes it difficult for either to identify and reach out to the families of overweight children without the support of the other. 

Although originally designed to facilitate the identification of overweight children, requiring enrollment forms or placing the services within a larger disease management program created barriers to utilization. Organizations that required an enrollment form found that many parents downloaded the forms from the website but never actually took their child to see the doctor and there was no way to track or contact the parent who downloaded the forms. The manual tracking and processing of forms required by some insurers also placed an administrative burden on providers.

#### 3.1.5. Marketing

Insurers tended to do more outreach to providers while employers engaged their employees through targeted mailings to those with children and email blasts. The marketing materials targeted to providers are distributed by email, intranet, direct mailing, and in-person. Marketing materials targeted to beneficiaries were distributed during the benefit open enrollment period by email, intranet, health fairs, direct mailing, and in-person. 

#### 3.1.6. Engaging Providers

The organizations took varied approaches to engaging providers. Three target providers participating in a primary care physician incentive plan while another is piloting the coverage in a single large pediatric practice. No organizations target specific dietitian groups. Respondents perceived that many providers are unaware of the coverage or do not know how to code visits appropriately to take advantage of the coverage. Several organizations reported that it is difficult to know if communications sent to providers ever actually reach them. 

Two of the three organizations with the highest usage rates are insurers who directly interacted with providers—sending network managers to meet directly with providers and go over a checklist of items including the obesity benefit and conducting in-service sessions about coding and the referral process. Four of the ten that directly contract with dietitians were concerned about an insufficient number of RDs in their network. Some organizations are developing alternative solutions such as allowing members to visit RDs at health departments or hospitals when contracted providers are not available in their area. Access to dietitians was seen as a particular challenge for rural areas. One insurer spent a year recruiting, credentialing, and contracting with RDs to build a referral network. At the same time, they had to educate RDs about how to work with medical practices and bill for services.

#### 3.1.7. Professional Organization Perspectives

 Staff from the AAP and Academy perceived that most pediatricians needed tailored practice strategies to facilitate the identification of overweight children and the creation of a referral network of dietitians, health educators, and fitness counselors to work with those children and families. More education around coding for obesity-related services and training sessions for office managers was also recommended, given the range of potentially appropriate codes and variability amongst insurers. Given that claims with obesity diagnoses have not been traditionally eligible for reimbursement most physician practices are not accustomed to documenting obesity or BMI status on billing forms. Similarly, it was felt that RDs would benefit from education about working with insurers, billing codes, and the processes required for reimbursement. 

#### 3.1.8. Engaging Families

Both employers and insurers use their benefits open enrollment period to introduce the coverage to members. Enrollment packets included a description of the obesity benefit and most signatories post information about the coverage on their websites and intranets. One employer sent email blasts to all employees who opted in with their email addresses. Some companies use targeted mailings that go only to members with children in the appropriate age range. Four organizations use claims data to identify children in families where a child and/or parent had been diagnosed with obesity and/or diabetes. One employer is using gift cards as incentives for members to complete their visits. 

### 3.2. Changes in Implementation over Time

#### 3.2.1. Benefit Changes

When asked whether any modifications had been made to implementation of the coverage, one insurer reported dropping the deductible for PCP and RD visits. Another removed the requirement to enroll in a disease management program. Both changes were made in an effort to increase participation.

#### 3.2.2. Engaging Beneficiaries

 Most organizations reported no change in their strategic marketing approach to beneficiaries, despite recognizing a need to increase member awareness of the coverage. Successful strategies that were reported include a video based on an employee who lost weight after receiving a Sports Authority gift card, a marketing campaign to members of a disease management program, and the publication of employee success stories in company newsletters. Privacy concerns prevent some organizations from sending targeted communications to obese children identified in claims data. One is offering a new wellness incentive to help motivate employees with those completing biometric screening eligible for a health plan premium discount. 

#### 3.2.3. Engaging Providers

Several organizations initiated new activities to engage providers. One insurer developed a provider training video on motivational interviewing which is available on the provider portal. Three organizations sponsored an educational webinar produced by the Alliance, AAP, and the Academy with CME credits. Most organizations felt that barriers remain. Two organizations find it challenging to promote the coverage to geographically dispersed provider groups and several noted that providers find it difficult to identify covered children when caring for patients covered by a range of insurance plans. Several organizations reported that providers continue to need education on the correct codes to bill for the services.

### 3.3. Use of Obesity-Specific Services

During the interviews, key informants reported that their internal data show low use of obesity-related services after one year of offering the coverage. Of the 10 organizations with the longest experience, several reported fewer than 50 children receiving counseling services and three reported between 100 and 1000 users with numbers increasing after the second year. Review of claims data from the organizations found that it was challenging to identify paid claims processed for obesity-related services. In order to track use, the provider must bill using a procedure or diagnosis code indicating that some type of weight-related education was performed (e.g., nutritional or exercise counseling). In order for administrators to be able to monitor service usage and patient outcomes, claims should also include the V85.5x diagnosis code that specifies the patient's BMI percentile. Based on review of claims paid, however, both physicians and dietitians used the less specific 278.0x diagnosis codes and counseling was rarely documented through diagnosis codes. The number of children with a documented diagnosis of obesity increased across the organizations after joining the initiative. The number of children with a diagnosis of obesity who had at least one preventive medicine visit also increased. The use of dietitian services varied widely and, not surprisingly, insurers that allowed direct billing by dietitians had the highest rates of dietitian use. 

## 4. Discussion

This study describes the early challenges of a unique, voluntary initiative to expand health insurance coverage in the United States for childhood obesity prevention and treatment services. These findings are relevant to many preventive health goals and can be used to improve the effectiveness of future efforts to promote preventive care through insurance redesign. The goal of the initiative to expand health care coverage for obesity services has been successful with all participating organizations able to implement and offer the coverage. Translating access into utilization, however, has been more challenging and participants identified several potential barriers that have impeded the increased utilization of obesity-related services. Making providers and families aware of new coverage for obesity services is challenging. Insurers have focused on making information available to providers using existing provider portals while employers have focused on outreach to their employees, particularly during open enrollment periods. From the provider perspective, varying requirements for documentation and varying acceptance of billing codes across insurers increase the complexity for providers who care for patients covered by a range of insurance plans. Precertification or enrollment requirements may allow insurers to identify and reach out to overweight children, but at the same time they increase the burden on families, thus discouraging participation. Copayments for primary care and dietitian visits varied widely and they discourage return visits because the copayment is applied to each individual visit. Finally, low use of BMI-specific diagnosis codes and counseling billing codes makes it difficult for organizations to monitor whether obesity prevention and counseling services are being used.

Development of effective and feasible strategies for prevention and treatment of childhood obesity in primary care settings is critical [15, 16]. Attitudinal and informational barriers are exacerbated by system barriers that make it difficult to provide proactive prevention and counseling services [17]. Previous studies of Medicaid programs have found that although the existing standards for Early and Periodic Screening Diagnostic and Treatment (EPSDT) allow coverage for comprehensive obesity-related care, only 11 states covered obesity-related nutritional and behavioral therapies and many created barriers to service delivery [18–20]. The Healthier Generation Benefit was recently highlighted as a promising example of payment strategies for comprehensive obesity prevention and treatment services and the lessons learned here are directly applicable to the expansion of first dollar coverage for approved preventive services under the Affordable Care Act [14]. 

This study has several limitations that must be noted. First, the implementation findings are dependent upon self-report and were not independently confirmed. To maximize accurate reporting, the interviews were performed and coded by the independent evaluation team. Also, the utilization data was independently analyzed by the evaluation team and the reported rates of utilization were generally confirmed. Secondly, the participating organizations joined the initiative voluntarily and may not be representative of private health insurers and employers nationwide.

The number of insurers and employers offering the Healthier Generation Benefit is growing with five new organizations joining the initiative in 2012. The early findings reported here are being used to assist new organizations in maximizing the impact of offering coverage. The AAP and Academy are collaborating on a provider outreach campaign. The campaign includes speaking engagements, webinars with continuing medical education credit, tools to make it easier to identify eligible beneficiaries, and an accurate coding campaign to encourage coding practices in line with AAP and USPSTF recommendations. Pilot projects are focusing on partnerships with specific providers to develop effective models for care coordination and patient outreach. Organizations continue to monitor utilization through claims data analysis and share best practices. Both reaching and engaging families are critical to long-term success. There is a clear need for more research exploring effective messaging to families about childhood obesity and promoting the use of available counseling resources. 

In sum, this study reports on the early findings from a unique initiative to expand childhood obesity prevention and treatment services through a voluntary partnership. The growing interest and participation in the initiative point to a recognition on the part of employers and insurers that the complicated problem of childhood obesity must be addressed. Reducing financial barriers to appropriate screening and prevention services is a necessary but not sufficient component of what by necessity must be a multifactorial approach. The experiences of these early adopters provide important lessons that can be used to guide the use of insurance incentives to promote broad public health goals in a decentralized health care system.

## 5. Conclusion

This paper reviews the lessons learned from a voluntary initiative to expand private health care coverage for childhood obesity prevention and treatment services. Large variability in implementation, best practices, and significant barriers were identified.

- The findings are relevant to many preventive health goals and can be used to improve the effectiveness of future efforts to promote preventive care through insurance redesign.
- Making providers and families aware of new coverage is challenging.
- Varying requirements for documentation and varying acceptance of billing codes across insurers increase the complexity for providers.
- Low use of BMI-specific diagnosis codes and counseling billing codes makes it difficult for organizations to monitor whether obesity prevention and counseling services are being received.

Reducing financial barriers is a necessary but not sufficient component of efforts to increase the utilization of obesity-related services. 

## Figures and Tables

**Table 1:** Key informant interview domains with sample questions.

Domain	Sample questions
Process of offering the coverage	Please describe the process used to roll out the new coverage in your organization
	(1) What steps are involved in launching it?
	(2) What steps have you completed?
	(3) Who is leading/led the initiative?
	(4) Who structured the roll-out process?
	(5) Please describe your roll-out process to providers
	(6) Please describe your roll-out process to eligible beneficiaries
	(7) What aspects of the rollout have gone well?
	(8) What aspects of the rollout have not gone well?
	(9) What, if any, additional barriers do you anticipate?

Enrollment process for families	According to your survey, there is (is not) some type of application/precertification process required.
	*Follow-up questions: *
	(a) If yes, please describe the process
	(b) Did your company have to create or modify any systems in order to handle this process? Please describe

Marketing efforts	(1) Have you received feedback from beneficiaries regarding your marketing/outreach activities?
	(a) If yes, what comments have you received?
	(2) Have you received feedback from your providers/employer groups regarding your marketing/outreach activities?
	(a) If yes, what comments have you received?

Engaging providers	(1) How do you regularly communicate with providers?
	(2) Are your dietitians a contracted provider?
	(3) Did you already have enough dietitians in the network, or did you have to contract more? Approximately how many?

Engaging families	In addition to the marketing materials described in your survey, please describe any additional approach(es) you are using to engage, inform, and/or educate families. (*Read through all prompts*)
	(a) Telephone prompts
	(b) Beneficiary incentives
	(c) Internet
	(d) Other

**Table 2:** Summary of participating organizations.

Type of organization	Geographic reach	Eligible children between ages 3 and 18
Health insurance plan	Pennsylvania	754,699
Health insurance plan	North Carolina	560,097
Health insurance plan	Virginia	488,423
Health insurance plan	Massachusetts	288,661
Health insurance plan	New York	86,010
Health insurance plan	Kentucky	46,225
Health insurance plan	National	30,400
Health insurance plan	Wisconsin	24,000
Health insurance plan	Wisconsin	212*
Health insurance plan	California	130*
Employer	National	53,000
Employer	National	20,175
Employer	Ohio	18,700
Employer	New York	9,018
Employer	National	1,256
Employer	National	624

*Targeting individual physician practices.

**Table 3:** Lessons learned from expansion of health insurance coverage.

Key processes	Findings
Claims processing	(i) Challenging to integrate BMI information with claims processing
	(ii) Insurer-specific billing codes are challenging for providers who bill to multiple insurers

Benefit structure	(i) Most offered more generous coverage than required by the initiative
	(ii) Most but not all allow direct billing by registered dietitians
	(iii) Copayments varied and were often significant

Coordination with other wellness programs	Programs often not coordinated

Enrollment requirements	(i) Pre-certification and disease management enrollment requirements created barriers to utilization
	(ii) Manual enrollment processes created barriers to provider participation

Marketing	(i) Insurers tend to focus outreach on providers
	(ii) Employers tend to focus outreach on employees

Engaging providers	(i) Difficult to know if materials ever reach the provider
	(ii) Direct interaction with providers is the most successful
	(iii) Insufficient number of registered dietitians available in some networks
	(iv) Providers have educational needs around motivational interviewing, billing codes, and effective care coordination between physician practices, dietitians, and health educators

Engaging families	(i) Most marketing efforts focused on open enrollment period
	(ii) Need for coordination between employers and insurers to effectively identify and reach overweight children

Monitoring utilization of obesity-related health services	(i) Infrequent use of BMI-specific billing codes
	(ii) Infrequent use of counseling-specific billing codes

BMI: body mass index.
